# Developmental Expression of IL-2-Receptor Light Chain (CD25) in the Chicken Embryo

**DOI:** 10.1155/1991/95302

**Published:** 1991

**Authors:** Barbara Fedecka-Bruner, Josef Penninger, Pierre Vaigot, Anne Lehmann, Carlos Martínez-A., Guido Kroemer

**Affiliations:** 1 Institut d'Embryologie du CNRS et du Collège de France 49bis avenue de la Belle Gabrielle F-94736 Nogent-sur-Marne Paris Cedex France; 2 Ontario Cancer Institute 500 Sherbourne Street Ontario Toronto M4X 1K9 Canada; 3 Centro de Biología Molecular CSIC, Universidad Autónoma Campus de Cantoblanco Madrid 28049 Spain

## Abstract

Thymocyte differentiation obeys the same fundamental principles in mammals as in avian species. This parallelism does not only affect the developmentally controlled acquisition of CD3, 4, 8, and TcR isotype expression, but also concerns CD25, the light chain of the interleukin-2 receptor (IL-2R). On chicken thymocytes, surface CD25, which is recognized by the monoclonal antibody INN Ch16, is first observed during day 11 of embryonic life, and peaks at day 14, when it is expressed by about one-third of all lymphoid cells. CD25 is found on subsets of all ,thymocyte populations as defined by TcRαβ, TcRγδ, 2, CD4, and CD8 expression, cortical or medullary localization, and is also present on a subset of intrathymic nurse-cell lymphocytes. These findings suggest phylogenetic conservation of the IL-2/IL-2R-triggered differentiation pathway previously described for mammalian species, thus under-lining its probable functional importance.


*Developmental Immunology*, 1991, Vol. 1, pp. 237–242
Reprints available directly from the publisher
Photocopying permitted by license only

© 1991 Harwood Academic Publishers GmbH
Printed in the United Kingdom

*Short Communication*

### Developmental Expression of IL-2-Receptor Light Chain (CD25) in the Chicken Embryo

BARBARA FEDECKA-BRUNER†, JOSEF PENNINGER§, PIERRE VAIGOT†, ANNE LEHMANN†, CARLOS MARTÍNEZ-A.‖, and GUIDO KROEMER*‖

†*Institut d'Embryologie du CNRS et du Collège de France, 49bis avenue de la Belle Gabrielle, F-94736 Nogent-sur-Marne, Paris Cedex, France*
§*Ontario Cancer Institute, 500 Sherbourne Street, Ontario M4X 1K9, Toronto, Canada*
‖*Centro de Biología Molecular, CSIC, Universidad Autónoma, Campus de Cantoblanco, 28049 Madrid, Spain*

Thymocyte differentiation obeys the same fundamental principles in mammals as in avian species. This parallelism does not only affect the developmentally controlled acquisition of CD3, 4, 8, and TcR isotype expression, but also concerns CD25, the light chain of the interleukin-2 receptor (IL-2R). On chicken thymocytes, surface CD25, which is recognized by the monoclonal antibody INN Ch16, is first observed during day 11 of embryonic life, and peaks at day 14, when it is expressed by about one-third of all lymphoid cells. CD25 is found on subsets of all thymocyte populations as defined by TcR$\alpha\beta$, TcR$\gamma\delta$, 2, CD4, and CD8 expression, cortical or medullary localization, and is also present on a subset of intrathymic nurse-cell lymphocytes. These findings suggest phylogenetic conservation of the IL-2/IL-2R-triggered differentiation pathway previously described for mammalian species, thus underlining its probable functional importance.

KEYWORDS: CD25, IL-2 receptor, thymus, T-cell ontogeny

### INTRODUCTION

During the past few years, intensive research activity has allowed to delineate the principles of thymic ontogeny as well as the successive differentiation states of thymus-dependent lymphocytes. According to current understanding, mesenchymal and epithelial elements interact with lymphocyte precursors either via cell contact or soluble mediators, thus propagating or arresting their proliferation and differentiation in terms of positive or negative selection processes (reviewed by Strominger, 1989; Blackman et al., 1990; von Boehmer and Kieselow, 1990). Although immunology owes the discovery of the fundamental dichotomy of the B- and T-cell systems to the chicken (*Gallus domesticus*) (Cooper et al., 1966), until recently the elucidation of avian T-cell differentiation was handicapped due to the paucity of suitable immunological reagents. The availability of monoclonal antibodies (mAbs) specific for the chicken equivalents of CD3 (Chen et al., 1986), CD4, and CD8 (Chan et al., 1988), as well as the T-cell receptor (TcR) $\alpha\beta$ and $\gamma\delta$ isotypes (Chen et al., 1988; Sowder et al., 1988), have recently allowed to characterize the phenotype of thymocytes in the course of embryogenesis. Thus, the first wave of T-cell development, from day 11 to day 14 of incubation (E11 to E14), is marked by an increasing population of predominantly CD3$^+$TcR$\gamma\delta^+$CD4$^-$CD8$^-$ cells. In a second phase, starting from E14, CD3$^+$TcR$\alpha\beta^+$ cells emerge, the majority of which acquire both CD4 and CD8 surface expression (Chan

*Corresponding author.

et al., 1988). TcR$\alpha\beta$ and TcR$\gamma\delta$ are expressed on a mutually exclusive basis (Chen et al., 1988). $CD3^+TcR\alpha\beta^+$ single positive ($CD4^+$ $CD8^-$ or $CD4^-CD8^+$) splenic or peripheral blood lymphocytes (PBL), $CD3^+TcR\gamma\delta^+$ double-negative ($CD4^-CD8^-$) PBL, and $CD3^+TcR\gamma\delta^+CD4^-CD8^+$ splenocytes become detectable after hatching (Chan et al., 1988; Chen et al., 1986).

Parallel to the characterization of the previously mentioned differentiation antigens, the description of lymphokines also has advanced in the chicken system. Thus, the discovery of chicken equivalents of interleukin-1 (IL-1) and interleukin-2 (IL-2) (Schauenstein and Kroemer, 1987), as well as the production of an mAb directed against the inducible light chain (CD25) of the heterodimeric high-affinity IL-2 receptor (Hála et al., 1986; Schauenstein et al., 1988) have demonstrated that peripheral T-cell activation obeys the same rules in the avian as in the mammalian immune system. In the present report, we addressed the question as to whether the involvement of IL-2 during thymocyte maturation, which is established for the human and the mouse system (Jenkinson et al., 1987; Shimonkevitz et al., 1987; Tentori et al., 1988; Pearse et al., 1989; Toribio et al., 1989; Zuñiga-Pflücker et al., 1990), is phylogenetically conserved. From the data presented herein, it will emerge that the expression of CD25 precedes the acquisition of "classical" T-cell markers in the chicken thymus and that CD25 is present on a quantitatively important minority of all T-cell subpopulations throughout ontogeny. These findings add to the conservation of principle cell-differentiation programs over large evolutionary intervals.

### RESULTS AND DISCUSSION

### CD25 Expression on Avian Thymocytes

INNCh16, which competetively inhibits the IL-2 response and the IL-2 adsorbtive capacity of activated T lymphocytes (Schauenstein et al., 1987), detects a membrane protein of an apparent molecular mass of 48–50 kD (Hála et al., 1986), that is, close to the 55 kD IL-2R light chain (CD25) of rodents or humans (Smith, 1989). As in mammals, CD25 is scarcely detectable on freshly isolated peripheral T cells, but is induced by short-term culture in the presence of T-cell mitogen (Kroemer et al., 1988; and Fig. 1). INNCh16 stains a significant portion of fetal thymocytes, although relatively weakly as compared to peripheral T lymphocytes activated *in vitro* with Concanavalin A (Fig. 1). Among these $CD25^+$ cells, both small and blastlike thymocytes are encountered.

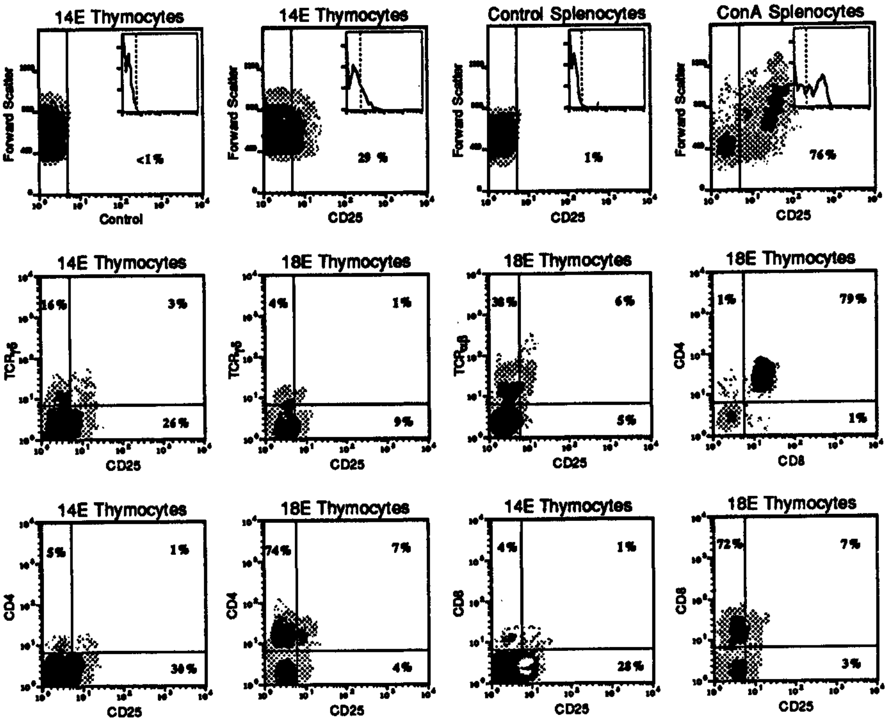

FIGURE 1. Cell-surface expression of CD25 on embryonic thymocytes. In the *upper panel,* FACS scattergrams and frequency distributions (*inserts*) of 14E thymocytes, fresh splenocytes derived from a 4-week-old animal, or splenocytes activated for 18 h with ConA stained with INNCh16 (specific for CD25) plus anti-IgM-FITC are shown. As a control, INNCh16 was omitted from the staining procedure. Note the elevated fluorescence intensity of $CD25^+$ ConA blasts as compared to thymocytes. In the *two lower panels,* FACS scattergrams of 14E or 18E thymocytes stained with INNCh16 plus anti-IgM-FITC in combination with monoclonal antibodies specific for TcR$\gamma\delta$ or TcR$\alpha\beta$ (biotin-labeled TcR1, TcR2; stained by means of PE-streptavidin conjugate), CD4 (CT4, PE-labeled), and CD8 (CT8, PE-labeled) are shown. CD4 (CT4, PE-labeled) and CD8 (CT8, TRITC-labeled) are coexpressed, whereas TcR$\gamma\delta$ and TcR$\alpha\beta$ are found on mutually exclusive subpopulations. Numbers indicate the percentage of cells found in the corresponding sector.

### Ontogenic Profile of CD25 Expression

As depicted in Fig. 2, CD25 expression in the thymus is first detectable on day 11 of embryonic life (11E), reaches its maximum at 14E, gradually declines until 20E, and peaks for a second time in the postnatal period. This kinetics contrasts with the monophasic transient IL-2R light-chain expression observed in the mouse fetus (Shimonkevitz et al., 1987) and can be speculatively related to the discontinuous thymic colonialization by lymphoid stem cells (Coltey et al., 1989). Both the kinetics and the high percentage of CD25 expression—up to one-third of all thymocytes—suggest the possibility that the majority of developing T lymphocytes transiently express CD25 during intrathymic maturation. Although at a much lower level, $CD25^+$ expression is also detectable on a minor fraction of the embryonic splenocytes (Fig. 2), among which no surface $CD3^+$ cells are detectable until hatching, but cytoplasmic $CD3^+$ surface $CD3^-TcR^-CD4^-CD8^+$ lymphocytes develop as a thymus-independent lineage (Bucy et al., 1989). These so-called TcR0 cells probably represent avian natural killer-cell equivalents (Bucy et al., 1990) and expand when the chicken embryo undergoes graft-versus-host reactions, where they express CD25 (Fedecka-Bruner et al., in preparation). In the posthatching phase, very few cells in the spleen, and virtually none in peripheral blood, express CD25 (Fig. 1).

### $CD25^+$ Thymocyte Populations

$CD25^+$ cells are first found in the thymic cortex (Fig. 3A). Corticomedullary transfer takes place before day 18, when medullary CD25 expression may be discerned (Fig. 3B). Double-staining experiments revealed the existence of $CD25^+$ cells within the double-positive ($CD4^+CD8^+$), double-negative ($CD4^-CD8^-$), $TcR\gamma\delta^+$, $TcR\alpha\beta^+$, and $TcR\alpha\beta^-\gamma\delta^-$ thymocyte subpopulations (Fig. 1, Table 1). $CD25^+$ 14E thymocytes are predominantly negative for T-cell markers, whereas 4 days later (18E), most $CD25^+$ cells belong to the double-positive stage, although the bulk of $CD4^+CD8^+$ thymocytes is $CD25^-$ (Table 1). Thus, CD25 expression in the chicken thymus shows an overall distribution analogous to that described in mammalian species with the only exception that in the chicken CD25 is expressed on a subset of double-positive thymocytes, which is not the case in mammals (Jenkinson et al., 1987; Toribio et al., 1988, 1989; Pearse et al., 1989). $CD25^+$ cells were encountered within a subpopulation ($24 \pm 5\%$) of intrathymic nurse-cell (TNC) lymphocytes (Figs. 3C and 3D). These cells might represent functionally active and relatively mature T lymphocytes, since INNCh16 blocks the capacity of isolated intra-TNC lymphocytes to mediate graft-versus-host reactions in the chorionallantoic membrane assay (Penninger et al., 1989, 1990).

### CONCLUDING REMARKS

In mammals, the IL-2/IL-2R system is thought to play a pivotal role in thymocyte proliferation and differentiation. Lymphoid cells committed to either the NK or T-cell lineages acquire the p70/75 heavy

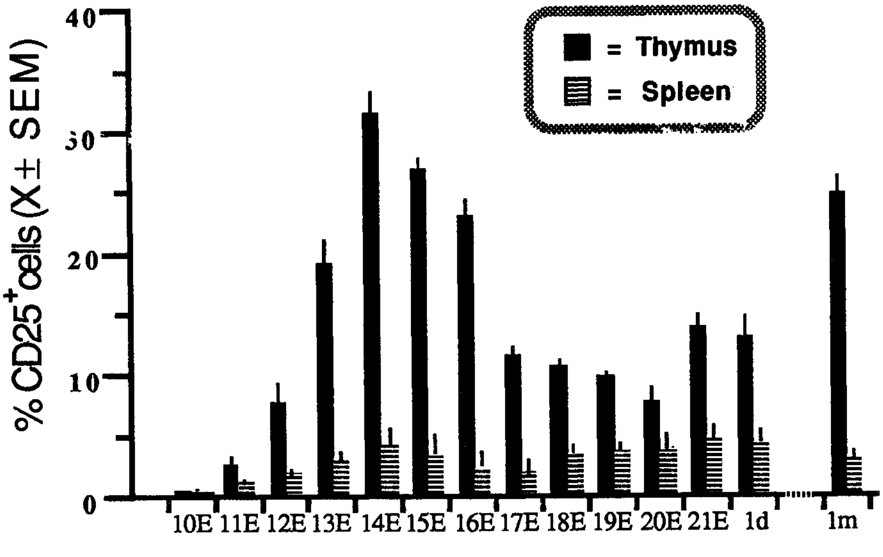

FIGURE 2. Ontogeny of CD25 expression in thymus and spleen. Cytofluorometric analysis was performed on cells stained with INNCh16 plus anti-IgM-FITC, as shown in Fig. 1. Splenocytes (striped columns) or thymocytes (black columns) were derived from 10- to 21-day-old embryos or from hatched animals. For each point, a minimum of five animals was analyzed. Cells were incubated with the vital dye acridin orange and positive cells were excluded from FACS analysis.

TABLE 1
Phenotype of Embryonic CD25$^+$ Thymocytes[a]

| Phenotype | % (X ± SEM) positives among | | | |
|---|---|---|---|---|
| | 14E | | 18E | |
| | Total thymocytes | CD25$^+$ cells | Total Thymocytes | CD25$^+$ cells |
| CD25$^+$ | 31 ± 3 | 100 | 11 ± 2 | 100 |
| CD4$^+$ | 5 ± 1 | 8 ± 2 | 83 ± 5 | 63 ± 5 |
| CD8$^+$ | 5 ± 2 | 9 ± 3 | 79 ± 4 | 64 ± 6 |
| CD4$^+$/CD8$^+$ | 5 ± 1 | 8 ± 2 | 82 ± 6 | 66 ± 4 |
| TcR$\alpha\beta^+$ | <2 | ND | 42 ± 3 | 49 ± 7 |
| TcR$\gamma\delta^+$ | 18 ± 3 | 9 ± 3 | 5 ± 2 | 15 ± 5 |
| TcR$\alpha\beta^+$/TcR$\gamma\delta^+$ | ND | ND | 49 ± 5 | 64 ± 5 |
| CD4$^+$/CD8$^+$CD4$^+$/TcR$\alpha\beta^+$/TcR$\gamma\delta^+$ | ND | ND | 86 ± 4 | 79 ± 6 |

[a]Thymocytes of 14- or 18-day-old embryos were stained with specific mAbs in combination with class-specific anti-IgM antiserum (FITC-labeled; for INNCh16, specific for CD25) or anti-IgG1-texas red conjugate (for all other mAbs) followed by cytofluorometric analysis. Mean values of at least three independent experiments are shown.

chain of the IL-2R (intermediate affinity IL-2R) on a prethymic stage. After migration into the thymus, interaction with stromal cells induces IL-2 and IL-2R light-chain (CD25) expression on pro-T cells (that still lack components of the CD3/TCR complex,

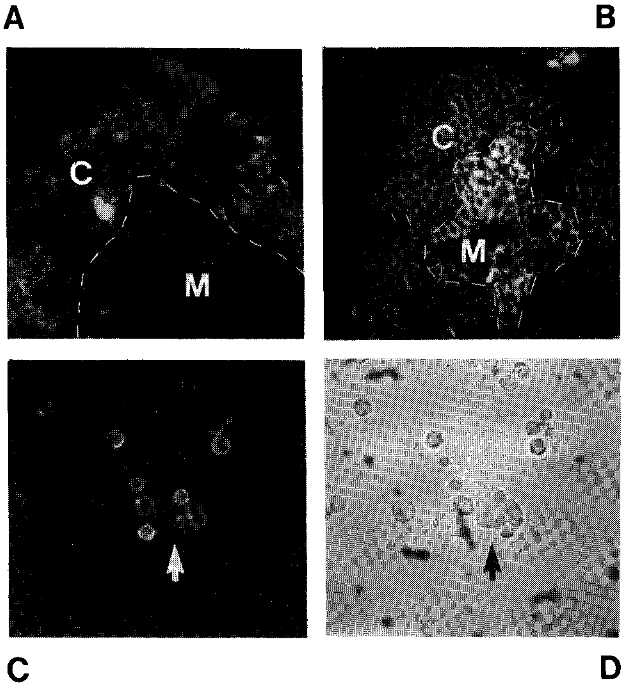

FIGURE 3. Histological analysis of CD25 distribution. Thymic sections of 14 (A, magnification 1×120) or 18-day (B, 1×300) old donors were stained with INNCh16 plus anti-Ig-FITC conjugate. The dashed line marks the boundary between thymic medulla (M) and cortex (C). In the lower part of the figure, the staining pattern of intrathymic nurse cell (TNC) lymphocytes (TNC marked by arrow) with INNCh16 (C) is compared to its trans-illumination picture (D).

CD4, and CD8), thus allowing formation of high-affinity heterodimeric IL-2R and autocrine proliferation via the IL-2/IL-2R pathway (Toribio et al., 1989). The inducibility of both IL-2 and CD25 is developmentally controlled, that is, is restricted to certain thymocyte populations (Boyer et al., 1989; Carding et al., 1989). Under *in vitro* culture conditions, IL-2 is capable of inducing CD3, TcR, CD4, and CD8 surface expression on immature thymocytes (Toribio et al., 1988). At the present stage, it is still a matter of debate whether the IL-2/IL-R-mediated autocrine pathway of T-cell differentiation is an abortive or a productive one, that is, whether such cells ultimately give rise to functional peripheral T cells. Other cytokines, including IL-1, IL-3, IL-4, IL-6, IL-7, TNF-$\alpha$, and GM-CSF, also have been implicated in thymic lymphopoiesis (reviewed by Martínez-A., 1990). Some findings have shed doubts on the obligatory involvement of the IL-2/IL-2R pathway in thymocyte maturation. In the human system, defects in IL-2 production by peripheral T cells have been observed that are not accompanied by major numeric T-cell deficiencies (Chatila et al., 1989; Disanto et al., 1990) and, in the rat, the expression of CD25 and IL-2 in double-negative cells has been contested (Takács et al., 1988). The fact that CD25 expression follows an analogous kinetics and distribution in the mammalian and the avian embryo suggests conservation of the IL-2-mediated thymocyte-differentiation pathway over about $270 \times 10^6$ years of independent evolution, thus underlining the probable functional importance of CD25 in thymic ontogeny. In this context, it is tempting to speculate that this early pro-T cell growth-promoting function of IL-2 might be the "original" one and that the involvement of IL-2 in peripheral T, NK, and B-cell physiology might have been acquired later during phylogeny. Investigations concerning more primitive vertebrate and invertebrate species might shed light on this issue.

### MATERIALS AND METHODS

### Animals and Cell Preparations

Thymi and spleens from normal White Leghorn chick (*Gallus domesticus*) embryos (eggs incubated at 38°C) or up to 4-week-old individuals were teased through 200-mesh sieves and cells suspended in ice cold PBS (pH = 7.2) supplemented with calf serum (10%). Viable cells were isolated by centrifugation of a Ficoll Hypaque$^R$ (Pharmacia, Uppsala, Sweden) gradient. In one experiment, splenocytes from 4-week-old donors ($5 \times 10^6$/ml) were cultured overnight in RPMI1640 (Seromed, Munich, FRG) supplemented with penicillin (100 U/ml), streptomycin (5 $\mu$g/ml), and concanavalin A (ConA, 2.5 $\mu$g/ml, Sigma, St. Louis, MO, USA) as described (Kroemer et al., 1988). Thymic nurse cells were isolated by Percoll$^R$ (Pharmacia) density-gradient centrifugation of collagenase type IV-digested thymic tissue and mounted on slides as described (Wick and Oberhuber, 1986).

### Immunocytohistochemistry

The mAbs INNCh16 (IgM, specific for CD25) (Hála et al., 1986), CT4 (IgG1, specific for CD4), CT8 (IgG1, specific for CD8) (Sowder et al., 1988), TcR1 (IgG1, specific for the $\gamma/\delta$ TcR) (Chen et al., 1986), and TcR2 (IgG1, specific for the $\alpha/\beta$ TcR (Chen et al., 1988) were used conjugated to phycoerythrin (PE) or fluorescein isothiocyanate (FITC) for direct fluorescence. For indirect staining, cells or tissue sections were incubated with unconjugated mAbs followed by FITC-conjugated antimouse IgM or texas red-conjugated antimouse IgG1 or with biotinylated mAbs followed by FITC or PE-conjugated streptavidin (Seromed, Blackthorn, UK). Automated flow cytometry was performed using the FACS II or FACS-Scan instruments (Becton Dickinson, Sunnyvale, CA). For monocolor fluorocytometry, cells were incubated with the vital dye acridin orange and stained cells were excluded from analysis. Results were expressed as percentage stained cells (mean values ± standard error of the mean, SEM) and significance was calculated using the Student t test.

### ACKNOWLEDGMENTS

The authors are indebted toward Dr. Cooper, Dr. Cihak, and Dr. Hála for the gift of valuable monoclonal antibodies; they also thank Dr. Dieterlen-Liévre for helpful discussion and Dr. LeDouarin for continuous support. This work was partially supported by grants from CICyT, FISS, EC, CNRS and by a fellowship of the Austrian Research Council (E.-S. 522Med to J.P.).

*(Received November 16, 1990)*

*(Accepted November 16, 1990)*

### REFERENCES

Blackman M., Kappler J., and Marrack, P. (1990). The role of the T cell receptor in positive and negative selection of developing T cells. Science **248:** 1369–1373.

Boyer P.D., Diamond R.A., and Rothenberg E.V. (1989). Changes in inducibility of IL-2 receptor $\alpha$-chain and T cell receptor expression during thymocyte differentiation in the mouse. J. Immunol. **142:** 4121–4130.

Bucy R.P., Coltey M., Chen C.H., Char D., LeDouarin N.M., and Cooper M.D. (1989). Cytoplasmic CD3$^+$ surface CD8$^+$ lymphocytes develop as a thymus-independent lineage in chick-quail chimeras. Eur. J. Immunol. **19:** 1449–1455.

Bucy R.P., Chen C.H., and Cooper M.D. (1990). Development of cytoplasmic CD3$^+$/T cell receptor-negative cells in the peripheral lymphoid tissues of chickens. Eur. J. Immunol. **20:** 1345–1350.

Carding S.R., Jenkinson E.J., Kingston R., Hayday A.C., Bottomly K., and Owen J.J.T. (1989). Developmental control of lymphokine gene expression in fetal thymocytes during T-cell ontogeny. Proc. Natl. Acad. Sci. USA **86:** 3342–3345.

Chan M.M., Chen C.H., Ager L.L., and Cooper M.D. (1988). Identification of the avian homologues of mammalian CD4 and CD8 antigens. J. Immunol. **140:** 2133–2138.

Chatila T., Wong R., Young M., Miller R., Terhost C., and Geha R.S. (1989). An immunodeficiency characterized by defective signal transduction in T lymphocytes. New Engl. J. Med. **169:** 696–702.

Chen C.H., Ager L.L., Gartland G.L., and Cooper M.D. (1986). Identification of a T3/T cell receptor complex in chickens. J. Exp. Med. **164:** 375–380.

Chen C.H., Cihak J., Lösch U., and Cooper M.D. (1988). Differential expression of two T cell receptors, TcR1 and TcR2, on chicken lymphocytes. Eur. J. Immunol. **18:** 539–543.

Coltey M., Bucy R.P., Chen C.H., Lösch U., Char D., LeDouarin M., and Cooper M.D. (1989). Analysis of the first two waves of thymic homing stem cells and their T cell progeny in chick-quail chimeras. J. Exp. Med. **170:** 543–557.

Cooper M.D., Peterson R.D.A., South M.A., and Good R.A. (1966). The functions of the thymus system and the bursa system in the chicken. J. Exp. Med. **123:** 75–86.

Disanto J.P., Keever C.A., Small T.N., Nichols G.L., O'Reilly R.J., and Flomenberg N. (1990). Absence of interleukin 2 production in a severe combined immunodeficiency disease syndrome with T cells. J. Exp. Med. **171:** 1697–1704.

Hàla K., Schauenstein K., Neu N., Kroemer G., Wolf H., Boeck G., and Wick G. (1986). A monoclonal antibody reacting with a membrane determinant expressed on activated chicken T lymphocytes. Eur. J. Immunol. **16:** 1331–1336.

Jenkinson E.M., Kingston R., and Owen J.J.T. (1987). Importance of IL-2 receptors in intra-thymic generation of cells expressing T cell receptors. Nature **329:** 160–162.

Kroemer G., Faessler R., Hàla K., Boeck G., Schauenstein K., Brezinschek H.-P., Neu N., Dietrich H., Jakober R., and Wick G. (1988). Genetic analysis of extrathyroideal features of Obese strain (OS) chickens with spontaneous autoimmune thyroditis. Eur. J. Immunol. **18:** 1499–1505.

Martínez-A. C., Ed. (1990). Interleukins and T cell development (Forum). Res. Immunol. **141:** 263–312.

Pearse M., Wu L., Egerton M., Wilson A., Shortman K., and Scollay R. (1989). A murine early thymocyte developmental sequence is marked by transient expression of the interleukin 2 receptor. Proc. Natl. Acad. Sci. USA **86:** 1614–1618.

Penninger J., Klima J., Kroemer G., Dietrich H., Hàla K., and Wick G. (1989). Intrathymic nurse cell lymphocytes can induce graft-versus-host reactions with high efficiency. Dev. Comp. Immunol. **13:** 313–327.

Penninger J., Hàla K., and Wick G. (1990). Intra-thymic nurse cell lymphocytes can induce a specific graft-versus-host reaction. J. Exp. Med. **172:** 521–529.

Schauenstein K., and Kroemer G. (1987). Avian lymphokines. *In:* Avian immunology: basis and practice, vol. 1, Toivanen A., and Toivanen P., Eds. (Boca Raton, FL: CRC Press), pp. 213–227.

Schauenstein K., Kroemer G., Hàla K., Boeck G., and Wick G. (1988). Chicken-activated-T-lymphocyte-antigen (CATLA) recognized by monoclonal antibody INN-CH 16 represents the IL-2 receptor. Dev. Comp. Immunol. **12:** 823–831.

Shimonkevitz R.P., Hussmann L.A., Bevan M.J., and Crispe I.N. (1987). Transient expression of IL-2 receptor precedes the differentiation of immature thymocytes. Nature **329:** 157–159.

Smith K.A. (1989). The interleukin 2 receptor. Annu. Rev. Cell Biol. **5:** 397–425.

Sowder J.T., Chen C.H., Ager L.L., Chan M.M., and Cooper M.D. (1988). A large subpopulation of avian T cells expresses a homologue of the mammalian T$\gamma/\delta$ receptor. J. Exp. Med. **167:** 315–322.

Strominger, J.L. (1989). Developmental biology of the T cell receptor. Science **244:** 943–950.

Takács L., Ruscetti F.W., Kovacs E.J., Rocha B., Brocke S., Diamantstein T., and Mathieson B.J. (1988). Immature, double negative (CD4−, CD8−) rat thymocytes do not express IL-2 receptors. J. Immunol. **141:** 3810–3818.

Tentori L., Longo D.L., Zuñiga-Pflucker J.C., Wing C., and Kruisbeek A.M. (1988). Essential role of the interleukin 2-interleukin 2 receptor pathway in thymocyte maturation in vivo. J. Exp. Med. **168:** 1741–1747.

Toribio M.L., Alonso J.M., Bárcene A., J.C, Guttiérrez de la Hera A., Marcos M.A.R., Márquez C., and Martínez-A. C. (1988). Human T-cell precursors: involvement of the IL-2 pathway in the generation of mature T cells. Immunol. Rev. **104:** 55–79.

Toribio M.L., Gutiérrez-Ramos J.C., Pezzi L., Marcos M.A.R., and Martínez-A. C. (1989). Interleukin-2-dependent autocrine proliferation in T cell development. Nature **342:** 82–85.

von Boehmer H., and Kieselow G. (1990). Self-nonself discrimination by T cells. Science **248:** 943–950.

Wick G., and Oberhuber G. (1986). Thymic nurse cells: a school for alloreactive and autoreactive cortical thymocytes? Eur. J. Immunol. **16:** 855–858

Zuñiga-Pflücker J.C., Smith K.A., Tentori L., Pardoll D.M., Longo D.L., and Kruisbeek A.M. (1990). Are the IL-2 receptors expressed in the murine fetal thymus functional? Dev. Immunol. **1:** 59–66.

